# Hypnotism or Psycho-Therapeutics

**Published:** 1891-03

**Authors:** 


### Reviews of Books.

---

*Hypnotism or Psycho-Therapeutics.* By R. W. Felkin, M.D. Edinburgh: Young J. Pentland. 1890.

This is a small book of 84 pages, a reprint of a series of articles contributed to the *Edinburgh Medical Journal.*

After a review of the various opinions that have been held in England of late years concerning hypnotism, the author proceeds to describe the means used to hypnotise persons, dividing these, as is usually done, into those acting by mental suggestion and those acting by physical stimulation. In doing so, he does not clearly point out that monotonous sensory stimulation, unaccompanied by any suggestion, produces only ordinary sleep; and that for hypnosis to occur, the person's attention must be fixed on the hypnotiser.

In describing the phenomena of hypnosis, Dr. Felkin gives, without a word of criticism, a description of Charcot's three stages—somnambulism, lethargy, and catalepsy—not even mentioning that these are now almost universally held to be cultivation-products—states, each characterised by a group of physical reflexes the result of education.

After a rather prolonged account of the possible dangers of hypnotism, Dr. Felkin gives a very fair account of the therapeutic uses of suggestion during hypnosis, showing that its field is practically limited to the treatment of non-organic nervous disorders; *e.g.*, neuralgias, apyretic muscular pain, habit spasms, stammering, insomnia, hysterical phenomena, morphinomania, etc.

There then follows a statement of the various theories as to what hypnosis is, and these are given at some length; but it is not pointed out what has been the general result—*i.e.*, that hypnosis has the greatest possible resemblance to ordinary sleep, only differing from it in that the attention is fixed on the hypnotiser, to the exclusion of modifying voluntary judgments and sensory impressions, so that all suggestions are accepted, and that on the cerebral changes corresponding to these depend the bodily effects observed.

The book is rather a collection of opinions than a critical account of the subject, and would have been more valuable a few years ago than at present; for we now have translations of the writings of Binet and Féré, Bernheim, Moll, and other authorities on the subject.

